# The Increased Frequency of Type 1 Regulatory T (Tr1) Cells and the Altered Expression of Aryl Hydrocarbon Receptor (AHR) and Interferon Regulatory Factor-4 (IRF4) Genes in Type 1 Diabetes: A Case-Control Study

**DOI:** 10.7759/cureus.65749

**Published:** 2024-07-30

**Authors:** Mohammed Sameir, Narjes Soleimanifar, Sara Assadiasl, Nihad Selman, Maryam Sadr, Hanieh Mojtahedi, Ali J Mohammed, Rasha H Abdulhussein, Zahraa M Hamid Al-Gawwam, Safin Hussein, Abdulmalik F Saber, Mohammad Hossein Nicknam

**Affiliations:** 1 Department of Clinical Autoimmune Therapy, Hammurabi College of Medicine, University of Babylon, Hilla, IRQ; 2 Molecular Immunology Research Center, Tehran University of Medical Sciences, Tehran, IRN; 3 College of Medicine, University of Babylon, Hilla, IRQ; 4 Department of Physiology, Hammurabi College of Medicine, University of Babylon, Hilla, IRQ; 5 Department of Pediatrics, Hammurabi College of Medicine, University of Babylon, Hilla, IRQ; 6 Department of Molecular Medicine, Tehran University of Medical Sciences, Tehran, IRN; 7 Department of Biology, University of Raparin, Ranya, IRQ; 8 Department of Psychiatry and Mental Health Nursing, College of Nursing, Hawler Medical University, Erbil, IRQ; 9 Department of Immunology, School of Medicine, Tehran University of Medical Sciences, Tehran, IRN

**Keywords:** regulatory t cells (tregs), interleukin 10 (il-10), interferon regulatory factor-4 (irf4), aryl hydrocarbon receptor (ahr), type 1 diabetes mellitus (t1dm)

## Abstract

Background and aim

Type 1 diabetes is an autoimmune disorder characterized by the destruction of pancreatic beta cells, leading to insulin deficiency and hyperglycemia. Regulatory T cells (Tregs), particularly type 1 regulatory T (Tr1) cells, play a crucial role in modulating autoimmune responses. Therefore, this study aimed to evaluate the frequency of Tr1 cells and their association with aryl hydrocarbon receptor (AHR) and interferon regulatory factor-4 (IRF4) gene expression levels in type 1 diabetes mellitus (T1DM) compared to the healthy controls.

Method

A case-control study design was used. The case group included patients diagnosed with T1DM, while the control group consisted of healthy individuals, matched for age and sex. Blood samples were collected, and peripheral blood mononuclear cells (PBMCs) were isolated. Serum interleukin 10 (IL-10) and interleukin 21 (IL-21) levels were measured using enzyme-linked immunosorbent assay (ELISA). The gene expression of AHR and IRF4 was analyzed using quantitative real-time polymerase chain reaction (qPCR), and Tr1 cell populations were determined using flow cytometry. Data were summarized with mean and standard error of the mean (SEM) for quantitative variables. Independent sample t-test, chi-square test, and the Mann-Whitney U test were used to compare groups. Statistical analyses were performed using SPSS version 25 (IBM SPSS Statistics, Armonk, NY), with significance levels set at p < 0.05. Figures were created using GraphPad Prism (GraphPad Software, San Diego, CA).

Results

A total of 45 cases were enrolled in the study, with 30 T1DM patients and 15 healthy controls. The mean IL-10 concentration was significantly higher in the patients (10.4 ± 1.1 pg/mL) compared to the healthy controls (5.1 ± 0.7 pg/mL), with a p-value of 0.001. There was no significant difference in IL-21 levels between the patients (76.1 ± 9.0 pg/mL) and healthy controls (88.2 ± 17.5 pg/mL), indicated by a p-value of 0.480. AHR gene expression was significantly lower in patients, with a p-value of 0.037. Although IRF4 gene expression was higher in patients, the difference was not statistically significant (p = 0.449). Tr1 cell frequency was significantly higher in T1DM patients (1.45% of cluster of differentiation 4+ {CD4+} T cells) compared to the healthy controls (0.40% of CD4+ T cells), with a p-value of 0.045.

Conclusions

The study demonstrated that T1DM is associated with higher IL-10 levels, decreased AHR gene expression, and a higher frequency of Tr1 cells. Policymakers should focus on developing targeted immunomodulatory therapies to address these immunological abnormalities. Healthcare providers should prioritize monitoring cytokine levels and gene expression in T1DM patients to tailor treatment plans effectively. Further research is needed to explore the therapeutic potential of modulating Tr1 cells and their related pathways in T1DM management.

## Introduction

Type 1 diabetes mellitus (T1DM) is a chronic autoimmune disorder characterized by the destruction of insulin-producing beta cells in the pancreas. This leads to a lifelong reliance on exogenous insulin and the potential development of various complications, including cardiovascular disease, neuropathy, and nephropathy [1]. T1DM affects a significant number of individuals globally, particularly children and adolescents, and its incidence is increasing [2]. Consequently, understanding the immune mechanisms involved in T1DM is of paramount importance in the development of effective therapeutic approaches.

Regulatory T cells (Tregs), specifically the type 1 regulatory T (Tr1) cell subset, are crucial in maintaining immune tolerance and preventing autoimmune diseases. Tr1 cells are known for their immunosuppressive properties, primarily through the secretion of interleukin 10 (IL-10) [3]. Understanding the functions and regulatory mechanisms of Tr1 cells is essential due to the autoimmune nature of T1DM. Recent research suggests that specific transcription factors, such as the aryl hydrocarbon receptor (AHR) and interferon regulatory factor-4 (IRF4), play a vital role in Tr1 cell differentiation and function. These factors could potentially be targeted to explore immune dysregulation in T1DM [4,5].

The significance of Tr1 cells in T1DM lies in their ability to modulate the immune response and maintain immune homeostasis. AHR is a ligand-activated transcription factor involved in various biological processes, including immune responses and detoxification [6]. It plays a critical role in Tr1 cell differentiation and the modulation of their suppressive functions [7]. The activation of AHR enhances Tr1 cell development, bolstering their production of IL-10 and their ability to suppress immune responses [8]. Similarly, IRF4 is a transcription factor essential for the development and function of various immune cells, including Tregs [9]. IRF4 is indispensable for IL-10 production in Tr1 cells, and its expression is crucial for their suppressive capacity [5]. The interplay between AHR and IRF4 in the regulation of Tr1 cells underscores a complex mechanism that may be disrupted in T1DM, potentially resulting in compromised immune regulation and the onset of autoimmunity.

Numerous investigations have been conducted to explore the role of Tregs in T1DM. These studies have consistently noted a decrease in the frequency and function of Tregs among T1DM patients compared to individuals without the condition [10,11]. However, there remains a lack of research concerning the dynamics of a specific Treg subset known as Tr1 cells, as well as their connection to the expression levels of AHR and IRF4. Evidence suggests that individuals with T1DM demonstrate an altered immune profile, characterized by diminished numbers and impaired function of Tregs [12]. This dysregulation extends to the Tr1 cell subset, which is essential for immune tolerance through its IL-10-mediated suppression of effector T cells [13]. A comprehensive investigation into the expression levels of AHR and IRF4 within Tr1 cells may provide valuable insights into the molecular mechanisms underlying T1DM and identify potential therapeutic targets. Understanding how these transcription factors affect Tr1 cell function may lead to the development of novel strategies for enhancing immune regulation in T1DM patients.

Despite the growing body of evidence highlighting the importance of Tregs in T1DM, there are still significant gaps in our understanding of the specific role of Tr1 cells and the molecular pathways that govern their function. Most studies conducted so far have focused primarily on the general population of Tregs, paying less attention to the Tr1 cell subset and its regulatory mechanisms. Furthermore, the potential interaction between AHR and IRF4 in regulating Tr1 cells within the context of T1DM has not been fully established. Addressing these gaps is crucial for obtaining a comprehensive understanding of the immunological deficits in T1DM and identifying new therapeutic targets. Current research efforts have primarily focused on the systemic effects of T1DM on immune function, with limited exploration of the specific transcriptional networks involved in regulating Tr1 cells. Therefore, our study aims to assess the frequency of Tr1 cells and their association with the expression levels of AHR and IRF4 genes in individuals with T1DM compared to the healthy controls.

## Materials and methods

Study design and subjects

A case-control design was employed for this study, which was conducted between 2023 and 2024. The case group included 30 patients diagnosed with T1DM, while the control group consisted of 15 healthy individuals. The control subjects were selected to match the case group in terms of age and sex and did not show any signs or symptoms of inflammatory diseases. Both the patients and healthy controls were between the ages of 12 and 19. The patients with T1DM who had other coexisting diseases were excluded from the study. Additionally, the control subjects had no personal or family history of T1DM or any autoimmune and inflammatory diseases.

Ethical considerations

The study protocol followed the guidelines established by the institutional research committee and was conducted in accordance with the Helsinki Declaration and similar ethical standards. Ethical approval was obtained from the institutional research committee of Tehran University of Medical Sciences, with the approval code IR.TUMS.CHMC.REC.1398.055. The parents or legal guardians of minors, as well as other participants, were required to sign informed consent forms before participating in the study.

Sample collection and processing

Blood samples were collected from both the case and control groups. To isolate peripheral blood mononuclear cells (PBMCs), each blood sample was diluted 2:1 with sterile phosphate-buffered saline (PBS). PBMCs were then separated using Ficoll-Hypaque centrifugation. After separation, the PBMCs were washed twice with PBS and resuspended.

Serum preparation and cytokine analysis

The serum was separated from whole blood by centrifugation, and the supernatant was collected. The enzyme-linked immunosorbent assay (ELISA) process was conducted using the Solarbio Immunoassay Kit (Beijing, China), which is a quantitative sandwich enzyme immunoassay technique for measuring human IL-10 and IL-21.

Gene expression analysis

RNA extraction and complementary DNA (cDNA) synthesis were performed using AddPrep Total RNA Extraction Kit (AddBio, Linköping, Sweden) and AddScript cDNA Synthesis Kit (AddBio, Linköping, Sweden) according to the manufacturer's instructions. The expression levels of AHR and IRF4 were measured using specific primers (Table 1) and real-time PCR techniques. The glyceraldehyde 3-phosphate dehydrogenase (GAPDH) gene was used as an internal control for the gene expression analysis.

**Table 1:** Primer sequences used in this study AHR, aryl hydrocarbon receptor; IRF4, interferon regulatory factor-4; GAPDH, glyceraldehyde 3-phosphate dehydrogenase

Gene	Primer Direction	Sequence
GAPDH	Forward	5'-GTCTCCTCTGACTTCAACAGCG-3'
	Reverse	5'-ACCACCCTGTTGCTGTAGCCAA-3'
AHR	Forward	5'-AGCAAGTTCACATGGAGGCA-3'
	Reverse	5'-CGTGGCAGCACCCTTTCTAT-3'
IRF4	Forward	5'-CTACACCATGACAACGCCTTACC-3'
	Reverse	5'-GGCTGATCCGGGACGTAGT-3'

Flow cytometry

The resuspended cells were stained with specific monoclonal antibodies (mAbs) obtained from Solarbio. The fluorochrome antibody dyes used were cluster of differentiation 3 (CD3) conjugated with phycoerythrin (PE), CD4 with Alexa Fluor 405, CD49b with fluorescein isothiocyanate (FITC), CD279/programmed cell death protein 1 (PD1) with antigen-presenting cell (APC), and lymphocyte-activation gene 3 (LAG-3) with Alexa Fluor 647. The analysis was performed using a FACScan Flow Cytometer (BD, Franklin Lakes, NJ) equipped with CellQuest software (BD, Franklin Lakes, NJ) to determine the Tr1 population.

Statistical analysis

Data were summarized and reported with frequency and percentage for qualitative variables and with mean and standard error of the mean (SEM) for quantitative variables. Independent sample t-test, chi-square test, and the Mann-Whitney U test were used to compare the results between the case and control groups. Statistical analyses were performed using SPSS version 25 (IBM SPSS Statistics, Armonk, NY), with significance levels considered at p < 0.05. Figures were created using GraphPad Prism (GraphPad Software, San Diego, CA).

## Results

Demographic characteristics

A total of 45 responses were analyzed, with 30 cases and 15 healthy controls. The mean age of controls was 16.60 ± 1.92 years, while for cases, it was 16.77 ± 2.06 years, showing no significant difference (p = 0.98). Systolic blood pressure distribution indicated that four (26.7%) controls and four (13.3%) cases had lower than 110 mmHg, while nine (60.0%) controls and 25 (83.3%) cases were between 110 and 130 mmHg, with no significant difference (p = 0.20). Diastolic blood pressure showed no significant difference, with eight (53.3%) controls and eight (26.7%) cases having lower than 80 mmHg (p = 0.19). Residence distribution showed no significant difference, with 10 (66.7%) controls and 20 (66.7%) cases living in urban areas (p = 0.64). For further details, refer to Table 2.

**Table 2:** General demographic characteristics of type 1 diabetes patients and the healthy controls The healthy controls numbered 15 and type 1 diabetes patients numbered 30, making a total of 45 participants. Data are represented as N, %, and mean ± SD. Chi-square and independent sample t-tests were used for data analysis. Significance was set at p < 0.05 SD: standard deviation

Variable	Type of Variable	Group Classification		Test Statistics	P-value
		Controls Number (Percent)	Cases Number (Percent)		
Age group	12-13	1 (6.7)	2 (6.7)	χ² = 5.17	0.98
	14-15	4 (26.7)	7 (23.3)		
	16-17	4 (26.7)	7 (23.3)		
	18-19	6 (40.0)	14 (46.7)		
	Mean ± SD	16.60 ± 1.92	16.77 ± 2.06		
Gender	Male	7 (46.7)	14 (46.7)	t = 0.00	0.62
	Female	8 (53.3)	16 (53.3)		
Systolic blood pressure	Lower than 110	4 (26.7)	4 (13.3)	χ² = 7.98	0.20
	Between 110 and 130	9 (60.0)	25 (83.3)		
	Above 130	2 (13.3)	1 (3.3)		
	Mean ± SD	116.0 ± 11.98	115.3 ± 9.37		
Diastolic blood pressure	Lower than 80	8 (53.3)	8 (26.7)	χ² = 7.52	0.19
	Between 80 and 90	7 (46.7)	21 (70.0)		
	Above 90	0 (0.0)	1 (3.3)		
	Mean ± SD	75.00 ± 10.18	76.33 ± 10.25		
Body mass index	Lower than 18	2 (13.3)	5 (16.7)	χ² = 4.53	0.96
	Between 18 and 25	10 (66.7)	19 (63.3)		
	Above 25	3 (20.0)	6 (20.0)		
	Mean ± SD	22.49 ± 3.50	21.56 ± 3.79		
Residence	Urban	10 (66.7)	20 (66.7)	t = 0.00	0.64
	Rural	5 (33.3)	10 (33.3)		

Diabetes-specific characteristics of T1DM patients

A total of 30 cases were analyzed, focusing on various clinical and demographic variables. Among the cases, two (6.7%) were smokers, and 19 (63.3%) had a significant medical history. The duration of illness varied, with 13 (43.3%) cases each in the 0-24-month and 25-48-month categories. C-peptide levels showed that 13 (43.3%) cases were in the 0.60-0.91 ng/mL range. Fasting blood sugar levels ranged from 9.10 to 19.50 mmol/L, with a mean of 13.67 ± 3.05 mmol/L. Glycosylated hemoglobin (HbA1c) levels had a mean of 9.92 ± 1.69 mmol/mol, with 13 (43.3%) cases in the 8.90-10.70 mmol/mol range. All cases were free of celiac or other diseases. Insulin doses varied, with a mean of 45.60 ± 24.05 IU, and the most common insulin type was soluble + neutral protamine Hagedorn (NPH), used by 13 (43.3%) of the cases (Table 3).

**Table 3:** Diabetes-specific characteristics of type 1 diabetes patients Type 1 diabetes patients numbered 30. Data are represented as N, %, and mean ± SD SD, standard deviation; NPH, neutral protamine Hagedorn; HbA1c, glycosylated hemoglobin

Variable	Type of Variable	Cases Number (Percent)
Smoking	Yes	2 (6.7)
	No	28 (93.3)
History	Yes	19 (63.3)
	No	11 (36.7)
Duration (months)	0-24	13 (43.3)
	25-48	13 (43.3)
	49-72	3 (10.0)
	73-96	1 (3.3)
C-peptide (ng/mL)	0.60-0.91	13 (43.3)
	0.92-1.14	10 (33.3)
	1.15-1.48	7 (23.3)
	Mean ± SD	0.99 ± 0.21
Fasting blood sugar (mmol/L)	9.10-11.90	11 (36.7)
	12.0-14.70	9 (30.0)
	15.0-19.50	10 (33.3)
	Mean ± SD	13.67 ± 3.05
HbA1c (mmol/mol)	6.90-8.80	8 (26.7)
	8.90-10.70	13 (43.3)
	10.80-13.60	9 (30.0)
	Mean ± SD	9.92 ± 1.69
Insulin dose (IU)	20-50	19 (63.3)
	51-80	8 (26.7)
	81-100	2 (6.7)
	101-120	1 (3.3)
	Mean ± SD	45.60 ± 24.05
Insulin type	Mixtard	7 (23.3)
	Actrapid	0 (0.0)
	NPH	4 (13.3)
	Soluble + NPH	13 (43.3)
	Soluble + Mixtard	5 (16.7)
	Apidra + Mixtard	1 (3.3)

Serum IL-10 and IL-21 levels in T1DM patients and the healthy controls

**Figure 1:**
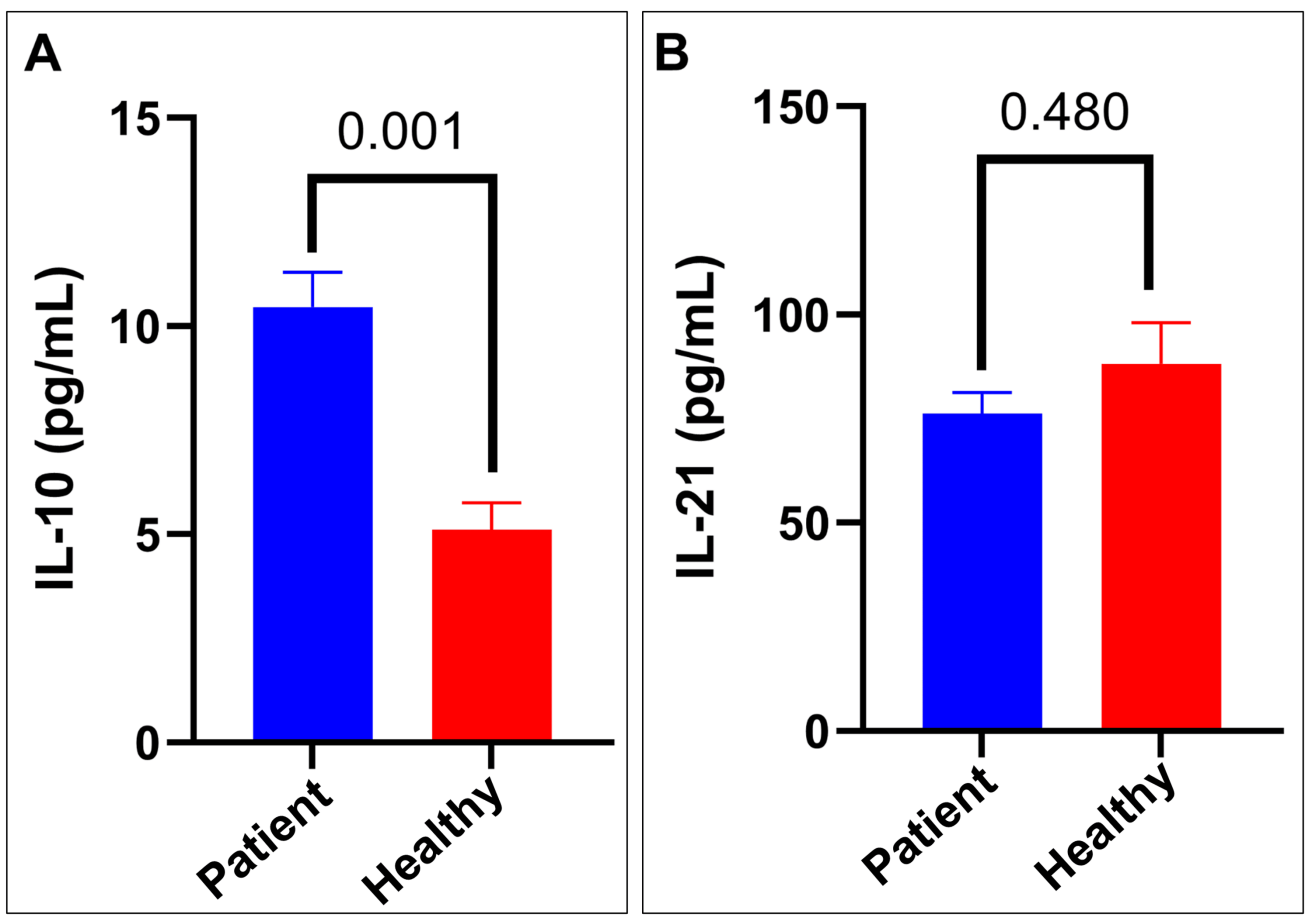
Comparison of IL-10 and IL-21 levels in healthy T1DM patients and controls (A) IL-10 concentration (pg/mL) in the patients and healthy controls. The patients show significantly higher IL-10 levels compared to the healthy controls (p = 0.001). (B) IL-21 concentration (pg/mL) in the patient and healthy groups. No significant difference (p > 0.05) in IL-21 levels between the two groups. Data are presented as mean ± SEM. Statistical significance was determined using the Mann-Whitney U test. Significance was set at p < 0.05 IL-10, interleukin 10; IL-21, interleukin 21; SEM, standard error of the mean; T1DM, type 1 diabetes mellitus

The analysis of serum IL-10 and IL-21 levels revealed different patterns in patients with T1DM compared to the healthy controls. Regarding IL-10, the patient group had a mean concentration of ~10 pg/mL, which was significantly higher than the control group's mean concentration of ~5 pg/mL. The difference was confirmed to be statistically significant through the Mann-Whitney U test (p = 0.001). On the other hand, there was no significant difference in IL-21 levels between the groups. The patient group had a mean IL-21 concentration of ~76 pg/mL, while the control group had ~88 pg/mL (Figure 1).

These results suggest an altered IL-10 profile in the patients with T1DM, indicating potential changes in inflammatory and regulatory processes linked to the disease. However, there was no significant difference in serum IL-21 levels between the groups, possibly due to the considerable variability observed within both populations. The contrasting findings between IL-10 and IL-21 highlight the complex nature of cytokine regulation in T1DM and call for further investigation into their roles in disease pathogenesis.

Gene expression analysis of AHR and IRF4

**Figure 2:**
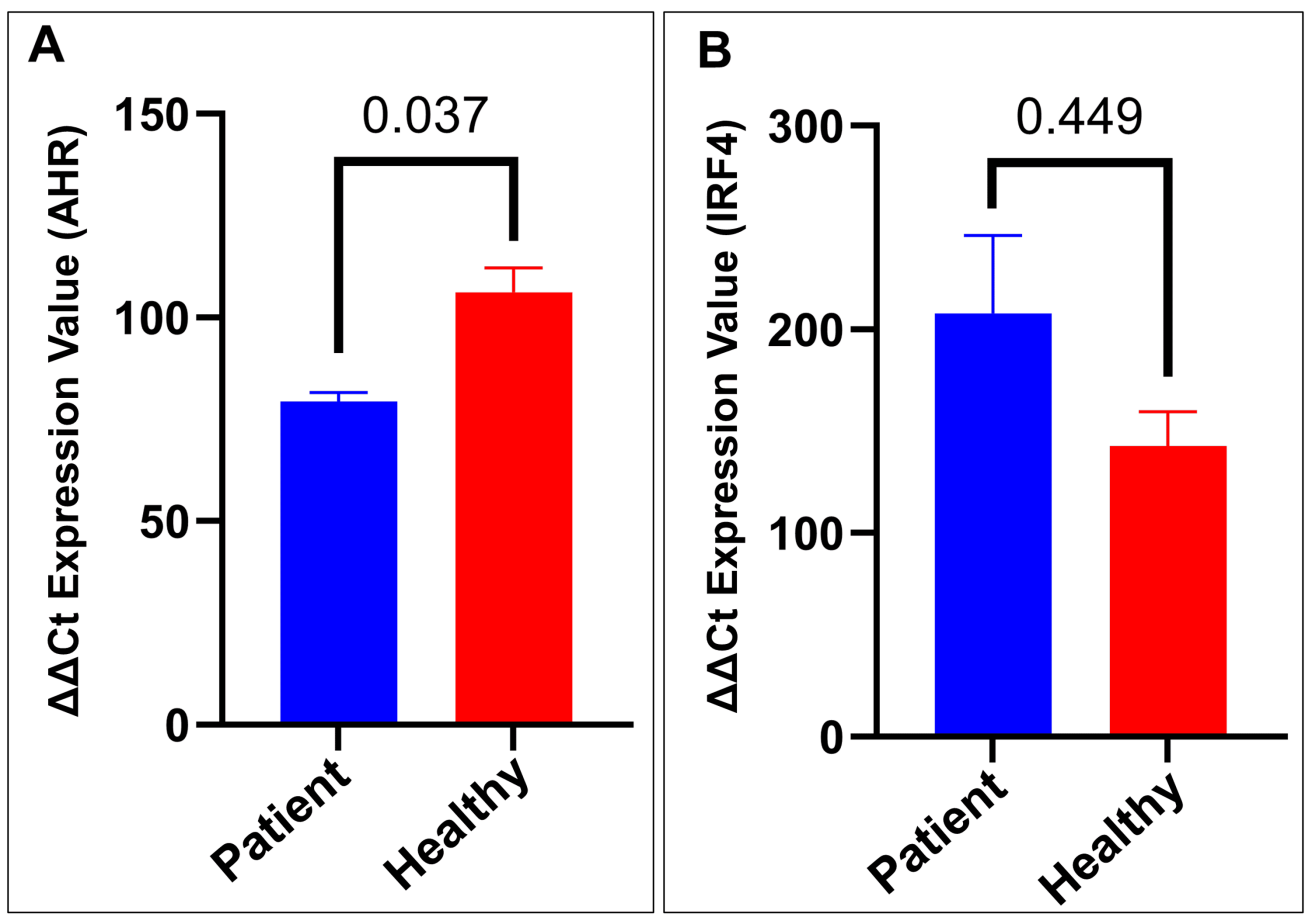
Expression of AHR and IRF4 in the healthy controls and T1DM patients (A) Relative delta-delta Ct expression value of AHR expression in healthy controls and the patients. The patients show significantly higher AHR expression compared to the healthy controls (p = 0.037). (B) Relative delta-delta Ct expression value of IRF4 expression in the healthy controls and patients. No significant difference (p > 0.05) in IRF4 expression between the two groups. Data are presented as mean ± SEM. Statistical significance was determined using the Mann-Whitney U test. Significance was set at p < 0.05 AHR, aryl hydrocarbon receptor; IRF4, interferon regulatory factor-4; SEM, standard error of the mean; T1DM, type 1 diabetes mellitus

The levels of AHR and IRF4 expression were analyzed in both the healthy controls and patients with type 1 diabetes mellitus. The analysis of AHR expression showed a significant difference between the patients and healthy controls (p = 0.037) (Figure 2A). The mean relative expression of AHR was lower in patients, suggesting the downregulation of AHR in patients with type 1 diabetes mellitus. Regarding IRF4, the analysis revealed a noticeable difference in expression levels between the patients and healthy controls, although this difference did not reach statistical significance (Figure 2B). Despite the lack of statistical significance, this trend suggests a potential upregulation of IRF4 in patients with type 1 diabetes mellitus. These findings indicate an altered expression of AHR and potentially IRF4 in patients with type 1 diabetes mellitus, which may have implications for the pathogenesis or progression of the disease.

Frequency of Tr1 cells in T1DM patients compared to the healthy controls

**Figure 3:**
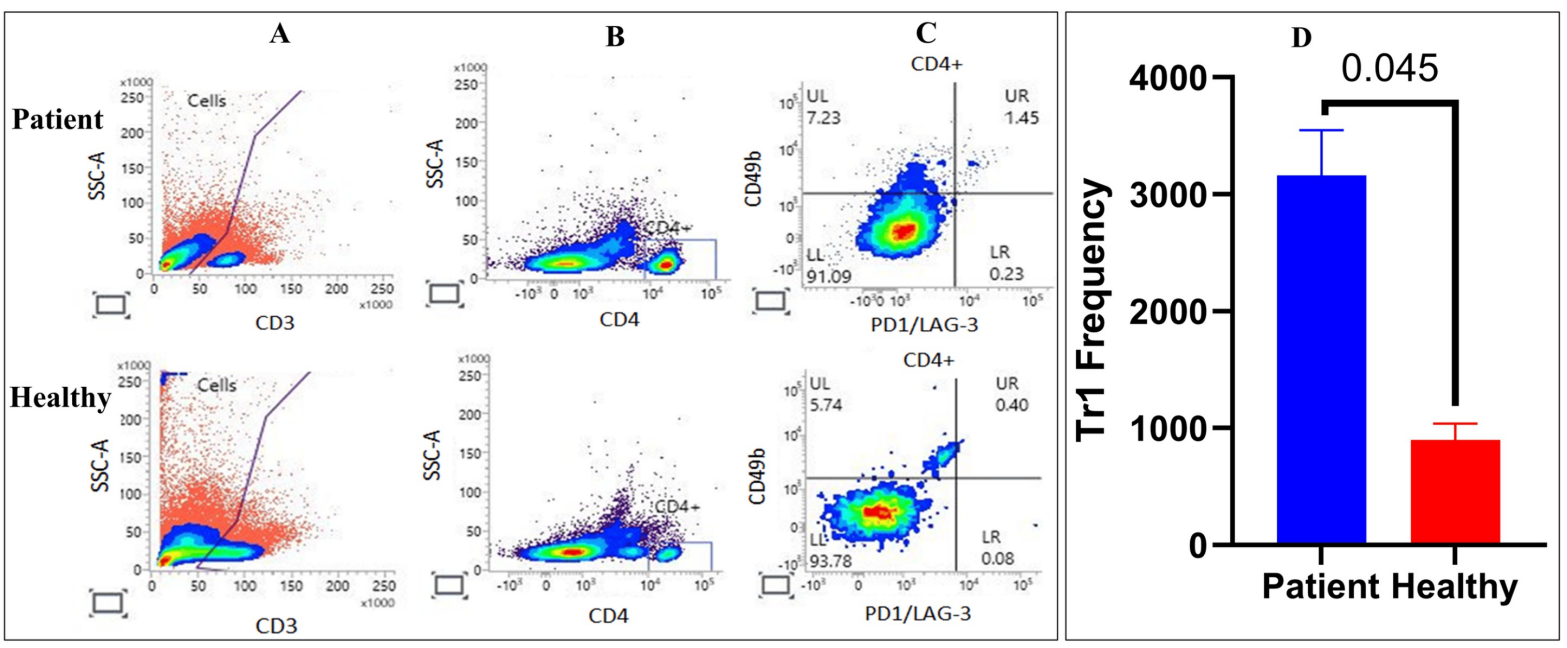
Flow cytometry analysis of Tr1 cells in PBMCs from T1DM patients and the healthy controls (A) Gating strategy for identifying lymphocytes based on SSC-A and CD3 expression. (B) The identification of CD4+ T cells within the CD3+ population. (C) The evaluation of CD49b and PD1/LAG-3 expression in CD4+ T cells. The upper right (UR) quadrant represents CD4+CD49b+PD1/LAG-3+ Tr1 cells. (D) Statistical significance was determined using the Mann-Whitney U test. Significance was set at p < 0.05 SSC, side scatter; T1DM, type 1 diabetes mellitus; PD1, programmed cell death protein 1; LAG-3, lymphocyte-activation gene 3; CD, cluster of differentiation; UL, upper left; LL, lower left; LR, lower right; PBMCs, peripheral blood mononuclear cells; Tr1, type 1 regulatory T cell

Flow cytometry analysis was conducted to determine the proportion of Tr1 cells in PBMCs from individuals with T1DM and healthy controls. The flow cytometry plots in Figure 3 demonstrate the approach used to identify Tr1 cells. In the healthy control sample, 0.40% of CD4+ T cells were categorized as CD49b+LAG-3+ Tr1 cells (Figure 3A). Conversely, the T1DM sample exhibited a higher frequency of Tr1 cells, with 1.45% of CD4+ T cells being Tr1 cells (Figure 3B). Tr1 cells were identified as CD3+CD4+CD49b+LAG-3+ cells (Figure 3C). Statistical analysis revealed a significant disparity (p = 0.045) in the frequency of Tr1 cells between T1DM patients and the healthy controls (Figure 3D). The average frequency of Tr1 cells was greater in T1DM patients compared to the healthy controls. These findings indicate an expansion of the Tr1 cell population in T1DM patients relative to healthy individuals.

## Discussion

The present study aimed to evaluate the frequency of Tr1 cells and their association with AHR and IRF4 gene expression levels in T1DM patients compared to the healthy controls. Overall, the results showed that the patients had a higher frequency of Tr1 cells and decreased expression of the AHR gene compared to the healthy controls. Additionally, there was a notable but not statistically significant increase in IRF4 gene expression in the patients.

Type 1 diabetes is an autoimmune disorder characterized by the destruction of pancreatic beta cells, leading to insulin deficiency and hyperglycemia. The role of Tregs, particularly Tr1 cells, in modulating autoimmune responses has gained increasing attention in recent years [14].

Understanding the mechanisms of Tr1 cells can help develop targeted therapies to improve patient outcomes. Tr1 cells produce immunosuppressive cytokines such as IL-10, which are crucial in maintaining immune tolerance. Additionally, AHR and IRF4 are vital in the differentiation and function of Tr1 cells, making them significant targets for investigation in the context of T1DM. To address these gaps, the study aimed to investigate the associations between Tr1 cells and AHR and IRF4 gene expression levels in type 1 diabetes patients compared to the healthy controls.

The demographic analysis revealed no significant differences in age, blood pressure, or residence distribution between the healthy controls and T1DM patients. This homogeneity in baseline characteristics strengthens the validity of the study's findings by minimizing potential confounding factors. However, the low prevalence of smoking and significant medical history among T1DM patients is noteworthy. Previous studies have reported associations between smoking and an increased risk of T1DM complications [15,16], highlighting the importance of addressing lifestyle factors in disease management.

The significant difference in serum IL-10 levels between T1DM patients and the healthy controls, with the patients showing higher concentrations, is an intriguing finding that warrants further discussion. IL-10 is generally considered an anti-inflammatory cytokine with immunoregulatory properties [17]. The elevated levels observed in T1DM patients could be interpreted as a compensatory mechanism to counteract ongoing autoimmune inflammation. This finding aligns with some previous studies that have reported increased IL-10 production in T1DM [18,19]. However, it contrasts with other research that has found decreased IL-10 levels in T1DM patients [20,21]. These discrepancies may be attributed to differences in disease duration, patient populations, or methodological approaches. The lack of a significant difference in IL-21 levels between T1DM patients and the healthy controls is somewhat surprising, given the known role of IL-21 in promoting T cell-mediated autoimmunity [22]. Previous studies have reported elevated IL-21 levels in T1DM patients and animal models [23,24]. The absence of a significant difference in our study could suggest that IL-21 regulation may vary depending on disease stage or genetic background. It also highlights the complex and sometimes contradictory nature of cytokine regulation in autoimmune diseases.

The analysis of gene expression revealed a significant difference in AHR gene expression between T1DM patients and the healthy controls. The patients showed lower expression levels, contradicting previous studies that have implicated AHR in the regulation of autoimmune responses [4,25]. The lower AHR expression in T1DM patients could indicate a compromised ability to promote Tr1 cell differentiation and function, potentially weakening a crucial protective mechanism against autoimmunity. This downregulation might contribute to the progression of the disease process, although the exact implications for T1DM pathogenesis require further investigation.

Interestingly, while IRF4 gene expression was higher in T1DM patients, the difference did not reach statistical significance. IRF4 plays a crucial role in Tr1 cell differentiation and function [5]. The trend toward higher IRF4 expression in T1DM patients, albeit not statistically significant, could suggest a partial activation of Tr1 cell-related pathways. The lack of statistical significance may be due to variability in IRF4 expression among patients or the influence of other regulatory factors.

Moreover, another key finding of this study is the flow cytometry analysis indicating a higher frequency of Tr1 cells in T1DM patients compared to the healthy controls. This expansion of the Tr1 cell population in the patients suggests an active immune regulatory response in T1DM. Previous studies have reported conflicting results regarding Treg frequencies in T1DM, with some showing decreased numbers [26,27] and others reporting no significant differences [28,29]. The increased Tr1 cell frequency observed in our study could represent a compensatory mechanism to counteract the ongoing autoimmune process. However, it raises questions about the functionality of these cells in the disease context. The expansion of Tr1 cells in T1DM patients, despite ongoing autoimmunity, suggests potential impairments in their suppressive capacity or the resistance of effector T cells to regulation. This phenomenon has been observed in other autoimmune diseases, where increased numbers of Tregs fail to control inflammation effectively [13]. Future studies should focus on assessing the functional properties of Tr1 cells alongside inflammatory biomarkers in T1DM patients to determine whether their increased frequency translates to enhanced immunoregulatory activity.

The examination of relationships between interleukin levels and gene expression revealed significant correlations between IL-10 levels and AHR expression in patients with T1DM. This finding supports the interconnected nature of cytokine production and gene regulation in the context of autoimmune responses. The negative correlation between IL-10 and AHR expression contradicts previous research demonstrating the role of AHR in promoting IL-10 production and the differentiation of Tr1 cells [30]. However, the complexity of these relationships is emphasized by the variability observed when analyzing these correlations alongside demographic characteristics. Factors such as disease duration, age, and smoking status may influence the strength and direction of these associations. This highlights the need for a personalized approach to understanding the pathogenesis of T1DM, taking into account individual patient characteristics and environmental factors.

The intricate interplay between cytokine levels, gene expression, and cellular frequencies observed in this study reflects the multifaceted nature of immune dysregulation in T1DM. While the increased frequency of Tr1 cells and elevated IL-10 levels suggest attempts at immune regulation, the persistence of autoimmunity indicates that these mechanisms are insufficient to fully control the disease process. Future research should focus on elucidating the functional capacity of Tr1 cells in T1DM and exploring potential therapeutic approaches to enhance their immunoregulatory effects.

Strengths and limitations of the study

This study provides quantitative, clinically relevant insights into immunological changes in T1DM, potentially linking Tr1 cell frequencies with AHR and IRF4 gene expression, which could contribute to a deeper understanding of the disease mechanisms and possible therapeutic approaches. The case-control design of this study limits our ability to observe changes in Tr1 cell frequencies and gene expression over time in T1DM patients. To gain a more thorough understanding of the dynamics of these immune parameters and their connection to disease progression, a longitudinal study that tracks the patients from diagnosis to various disease stages would be more informative.

## Conclusions

The study demonstrated that T1DM is associated with higher IL-10 levels, decreased AHR gene expression, and a higher frequency of Tr1 cells. Policymakers should focus on developing targeted immunomodulatory therapies to address these immunological abnormalities. Healthcare providers should prioritize monitoring cytokine levels and gene expression in T1DM patients to tailor treatment plans effectively. Further research is needed to explore the therapeutic potential of modulating Tr1 cells and their related pathways in T1DM management.
